# Multifunctional Nature of the Arenavirus RING Finger Protein Z

**DOI:** 10.3390/v4112973

**Published:** 2012-11-09

**Authors:** Sarah Katharina Fehling, Frank Lennartz, Thomas Strecker

**Affiliations:** Institut für Virologie der Philipps-Universität Marburg, Hans-Meerwein-Str. 2, 35043 Marburg, Germany; Email: fehling@staff.uni-marburg.de (S.K.F.), lennartz@students.uni-marburg.de (F.L.), strecker@staff.uni-marburg.de (T.S.)

**Keywords:** Arenavirus, ESCRT, Lassa virus, Lymphocytic choriomeningitis virus, Junin virus, matrix protein, RING finger protein, virus assembly and budding, virus-host cell interaction, Z protein

## Abstract

Arenaviruses are a family of enveloped negative-stranded RNA viruses that can cause severe human disease ranging from encephalitis symptoms to fulminant hemorrhagic fever. The bi‑segmented RNA genome encodes four polypeptides: the nucleoprotein NP, the surface glycoprotein GP, the polymerase L, and the RING finger protein Z. Although it is the smallest arenavirus protein with a length of 90 to 99 amino acids and a molecular weight of approx. 11 kDa, the Z protein has multiple functions in the viral life cycle including (i) regulation of viral RNA synthesis, (ii) orchestration of viral assembly and budding, (iii) interaction with host cell proteins, and (iv) interferon antagonism. In this review, we summarize our current understanding of the structural and functional role of the Z protein in the arenavirus replication cycle.

## 1. Introduction

**Figure 1:**
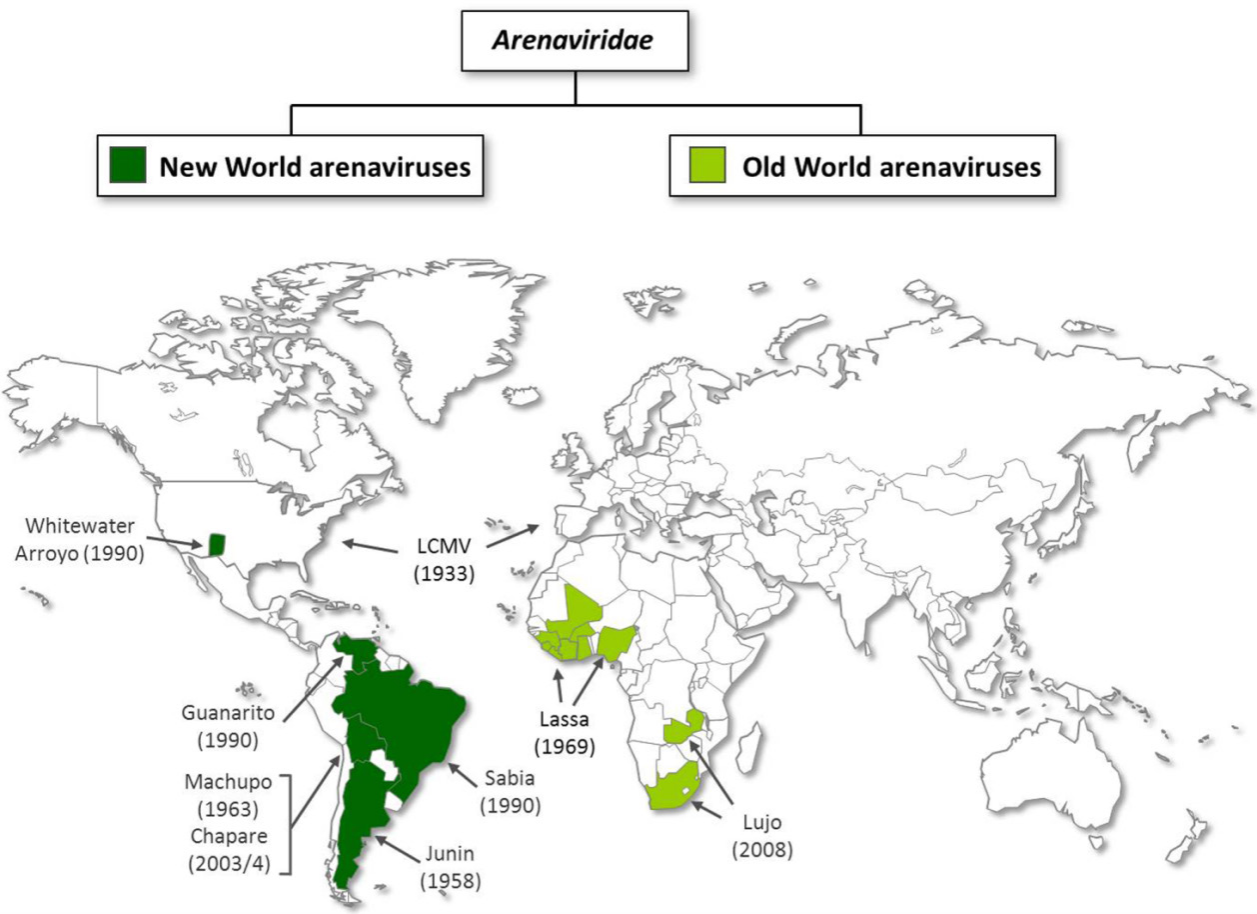
Geographic distribution of human pathogenic arenaviruses. This map summarizes the distribution of human pathogenic New and Old World arenavirus species. The year of the first description is indicated in brackets.

The *Arenaviridae* family consists of one unique genus (*Arenavirus*) that currently comprises 24 recognized virus species as defined by the International Committee on Taxonomy of Viruses. According to their antigenic properties, their serological relationships and their geographical distribution, arenaviruses have been classified into two groups: the New World (NW) and the Old World (OW) group (Figure 1). NW arenaviruses can be further divided into three clades (A, B, and C), based on the sequence of their nucleocapsid genes. The OW group includes the globally distributed prototypic arenavirus, lymphocytic choriomeningitis virus (LCMV), as well as the highly human pathogenic Lassa virus (LASV), which is the causative agent of the acute viral hemorrhagic Lassa fever found in West Africa; and Lujo virus (LUJV) that has been associated with severe hemorrhagic fever disease in five patients in southern Africa [1,2,3]. OW arenaviruses found throughout the African continent but not known to be pathogenic for humans are Ippy (IPPV), Mobala (MOBV), Mopeia (MOPV), Morogoro, and Kodoko viruses [4,5,6]. Among others, the NW complex comprises Junin virus (JUNV) in Argentina, Chapare (CHPV) and Machupo viruses (MACV) in Bolivia, Sabia virus (SABV) in Brazil, Tacaribe virus (TCRV) in Trinidad, Whitewater Arroyo virus (WWAV) in the United States, and Guanarito virus (GTOV) in Venezuela [7,8,9]. Five of the South American arenaviruses (CHPV, GTOV, JUNV, MACV, and SABV) are associated with hemorrhagic fever (HF) in humans and, together with LASV and LUJV, they make arenaviruses to the largest currently known family of HF-causing viruses.

The principal hosts of NW and OW arenaviruses are rodents, and the only exception to date is TCRV which was isolated from fruit-eating bats [10]. However, recent studies showed that TCRV causes fatal infection in Jamaican fruit bats (*Artibeus jamaicensis*), indicating that these may not be the natural reservoir host for TCRV [11]. The geographic distribution of each arenavirus is determined by the range of habitats of its reservoir species. LCMV is the only arenavirus known to be distributed worldwide due to its association with its wide-spread host *Mus musculus*. Transmission of arenaviruses from their respective hosts to humans is assumed to occur mainly through inhalation of particulates contaminated with infectious rodent excretions. In addition, hunting of peridomestic rodents and consumption of their meat as a protein source are recognized as potential risk factors for rodent-to-human transmission of LASV in certain regions of Guinea [12]. Direct contact with blood and body fluids or contaminated materials from highly viraemic patients significantly increases the risk of human-to-human transmission of HF arenaviruses during nosocomial outbreaks [13,14].

Among HF-causing arenaviruses, the known distribution of LASV covers the greatest geographical range, being endemic in several West African countries, with outbreaks most frequently observed in Guinea, Liberia, Nigeria, and Sierra Leone [15,16,17,18,19,20,21]. Virus isolation or serological evidence of human LASV infection has also been documented in the Central African Republic, Ivory Coast, Mali, and Senegal [22,23,24,25,26]. Therefore, LASV constitutes a significant public health burden, with more than 220 million people living at potential risk in these countries. Additionally, due to increased air travel and inter-regional travel, as well as international peacekeeping in areas of conflict in which LASV is endemic, LASV has been transported numerous times to Europe, North America, and Japan, making it responsible for the largest number of reported cases of imported viral hemorrhagic fevers (VHF) in industrial countries [27,28,29,30,31,32,33,34,35,36,37]. 

In spite of the threat that HF arenaviruses pose, the live attenuated JUNV vaccine known as Candid #1 is currently the only vaccine that has been found to be safe and highly efficacious against JUNV infection when tested in endemic areas [38], although progress in the development of effective vaccines for other arenaviruses has been reported [39,40]. The only existing drug used to treat Lassa fever and certain South American VHFs is the broad-spectrum antiviral agent Ribavirin, a ribonucleoside analog, that has been shown to be partially effective if administered in the early course of illness [41]. Due to the severe and often fatal outcome of infection, non-availability of vaccine prophylaxis, and inadequate therapeutic treatment options, HF arenaviruses are defined as Category A Priority Pathogens by the National Institute of Allergy and Infectious Diseases (NIAID), and are listed as Biosafety Level 4 (BSL-4) agents.

## 2. Virion architecture, gene organization and virus replication

**Figure 2:**
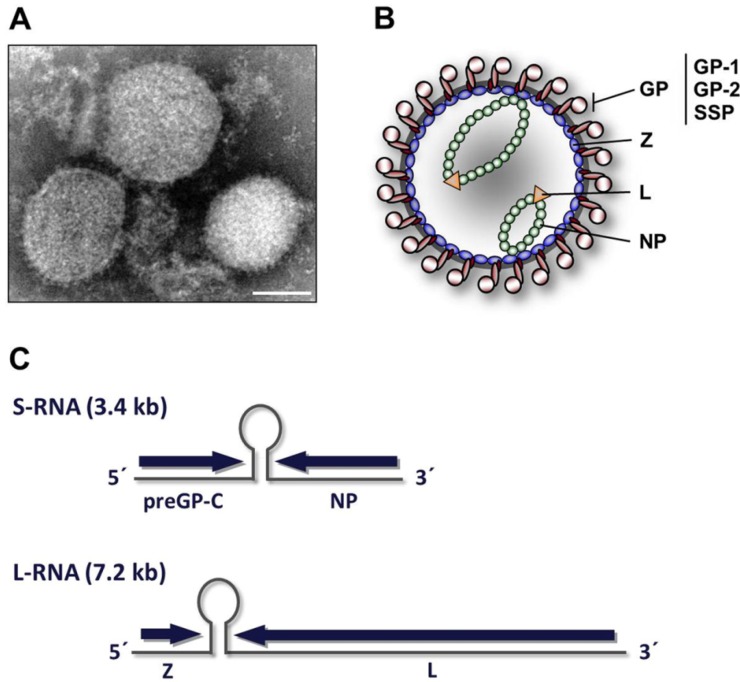
Arenavirus virion structure and genome organization. **(A)** Electron microscopic image of Lassa virus (LASV) illustrates the common virion architecture of arenaviruses. Bar, 100nm. **(B)** Schematic representation of arenavirus virions. The viral envelope, a lipid bilayer derived from the host cell plasma membrane, contains multiple copies of glycoprotein spikes on the surface that are required for receptor binding and virus entry. The Z protein forms a matrix layer underneath the viral membrane. The nucleoprotein NP associates with the polymerase L to form together with the genomic RNA the ribonucleoprotein (RNP) complex. **(C)** Genome organization of arenaviruses. Arenaviruses contain a bi-segmented negative-strand RNA genome, composed of the small (S) RNA segment and the large (L) RNA segment. Each RNA segment encodes two viral proteins in an ambisense orientation. The open reading frames are separated by intergenic regions.

Arenaviruses are lipid-enveloped viruses containing a bi-segmented negative-strand RNA genome. Each genomic RNA segment encodes two non-overlapping open reading frames in an ambisense orientation, separated by a non-coding intergenic region. The small (S) RNA encodes the nucleoprotein NP and the glycoprotein precursor preGP-C, which undergoes co- and post-translational cleavage events in order to achieve its mature form [42,43,44,45]. The large (L) RNA segment encodes the RNA-dependent RNA polymerase L and the small RING finger protein Z [46,47,48,49]. Arenavirus virions are spherical or pleomorphic and vary in diameter from 60 to 300 nanometers (Figure 2A). It is assumed that the lipid bilayer of the viral envelope derives from the host cell membrane where arenavirus budding occurs. The virion surface is covered with trimeric glycoprotein spike complexes [50,51]. Within the virion, a matrix layer composed of Z protein lines the inner leaflet of the membrane [51,52,53]. Solitary expression of Z protein is sufficient for the production of lipid-enveloped virus-like particles (VLPs) that are morphologically similar to virus particles released from infected cells [54]. The genomic RNAs are thought to assemble with L and NP proteins into ribonucleoprotein complexes (RNPs). A schematic representation of the virion architecture and geneorganization of arenaviruses is shown in Figure 2 (B and C).

For virus entry into host cells, OW arenaviruses and Clade C NW arenaviruses use α-dystroglycan as a cellular receptor [55,56], which normally functions as a transmembrane linkage between the actin-based cytoskeleton and the extracellular matrix. Although α-dystroglycan is presumably the main receptor for LASV and LCMV, recent findings suggested the existence of four alternative receptor molecules: DC-SIGN and LSECtin from the C-type lectin family, and the receptor tyrosine kinases Tyro3 and Axl [57,58]. However, the role these molecules play in cell-specific targeting and tissue tropism of LASV and other arenaviruses *in vivo* remains unclear. Clade B NW arenaviruses employ the transferrin receptor 1 (TfR1) as an entry receptor for the infection of target cells [59], while the cellular receptor for Clade A NW arenaviruses has not yet been identified. Upon receptor binding, NW arenaviruses enter the host cells through clathrin-mediated endocytosis [60], while the internalization route of OW arenaviruses is clathrin-independent [61,62,63]. Recent studies demonstrated that LASV and LCMV cell entry occurs through late endosomes/multivesicular bodies (MVBs). This novel arenavirus entry pathway is thought to be linked to the cellular trafficking and degradation route of α-dystroglycan [64]. The low pH environment of late endosomes is necessary for the virus-endosome membrane fusion triggered by the glycoprotein GP [65]. Following the release of the RNPs into the host cell cytoplasm, viral replication and transcription are initiated. 

During genome replication, a full-length, anti-genomic copy of the genomic S and L RNA is synthesized. The purified genomic and antigenomic RNA species alone are unable to direct the synthesis of viral polypeptides and thus are not infectious. Due to the ambisense coding strategy, both genomic and anti-genomic RNA serve as templates for transcription of viral mRNA. The transcripts contain a 5` cap but are not polyadenylated [66]. The first synthesized viral proteins are NP and L, which represent the minimal viral *trans*-acting factors required for efficient viral RNA synthesis [67,68]. GP-C and Z are transcribed from the anti-genomic RNA of the S and L segments. The anti-genomic RNA also serves as a template for the generation of genomic RNA segments, which, together with NP and L, associate to RNPs. Finally, the RNPs are packaged and progeny virions are released from the cell. The membrane derived from the host cell that envelopes the budding virus particles bears the envelope glycoprotein GP.

## 3. The multiple roles of Z protein in the arenavirus life cycle

**Figure 3:**
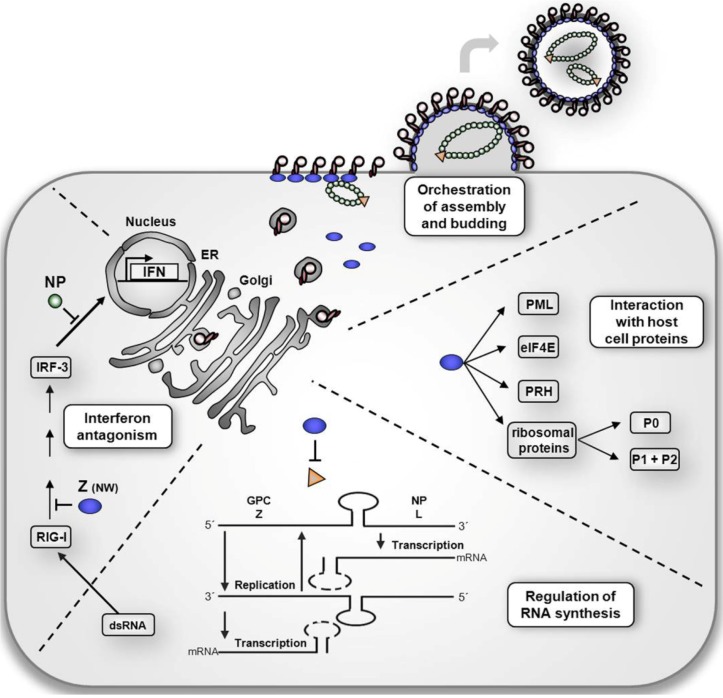
Functional roles of the arenavirus Z protein in the viral life cycle. **Regulation of viral RNA synthesis.** Transcription and replication of arenaviruses takes place in the host cell cytoplasm. Z protein regulates these processes through interaction with the viral polymerase. **Orchestration of viral assembly and budding.** Z protein drives particle release at the plasma membrane. Through interaction with GP and viral RNPs, Z mediates their incorporation into nascent virions. **Interaction with host cell proteins.** Z protein interacts with several host cell factors, such as PML (promyelocytic leukemia protein), eIF4E (eukaryotic translation initiation factor 4E), PRH (proline-rich homeodomain protein), and ribosomal P proteins. The assumed biological effects of these interactions on arenavirus replication are discussed in the text. **Interferon antagonism. **Z proteins from various NW arenaviruses participate in IFN antagonism.While Z protein inhibits the activation of RIG-I-mediated downstream signalling pathway through direct interaction with RIG-I, NP, which is the main arenavirus interferon antagonist, causes a block in the IRF-3 activation pathway and prevents nuclear translocation of IRF-3.

Due to their small genome size and small number of genes, arenaviruses evolved a powerful strategy for maintaining efficient virus replication by encoding multifunctional viral proteins that mediate several distinct virus-virus and/or virus-host protein-protein interactions. In particular, the smallest arenavirus gene product, the RING finger protein Z, has multiple functions and acts at various stages during viral infection. As illustrated in Figure 3, essential functions of the Z protein include (i) regulation of viral RNA synthesis, (ii) orchestration of viral assembly and budding, (iii) interaction with host cell proteins, and (iv) antagonizing the host cell interferon (IFN) system. In the following sections we will review the current knowledge on this remarkably multifunctional viral protein and discuss how the Z protein facilitates its various activities. 

**Figure 4:**
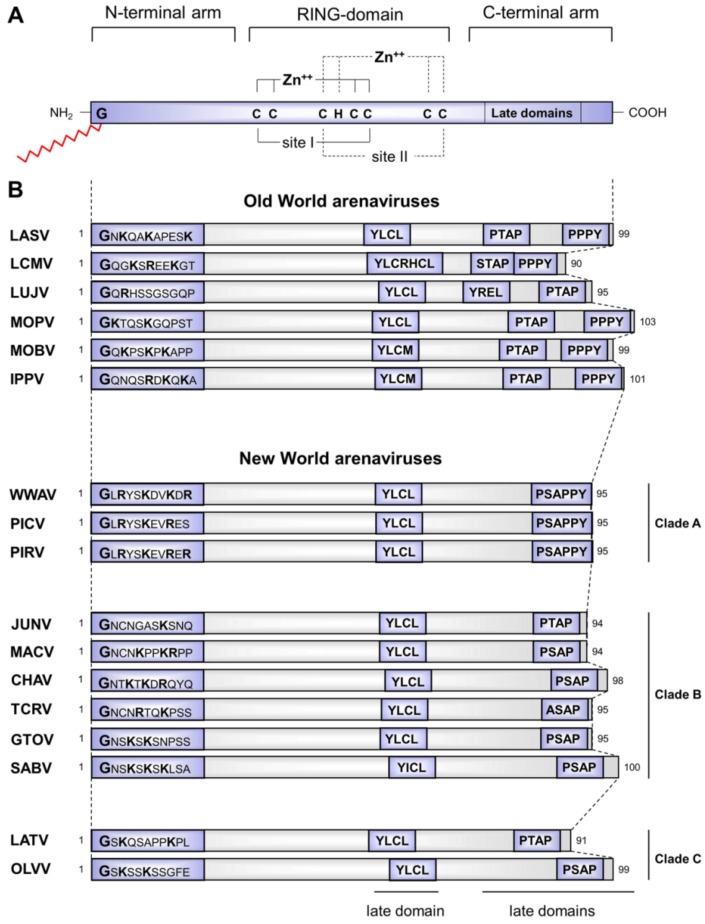
Schematic representation of the linear organization of arenavirus Z protein. **(A)** Domainstructure of the Z protein. The Z protein contains an N-terminal myristoylation site for attachment of myristic acid (shown in red), and a conserved, centrally located RING domain, which coordinates the binding of two zinc atoms. Structural characterization of LASV Z protein suggested that the N- (residue 1-29) and the C-terminal (residue 71-99) arms flanking the RING domain are highly flexible, enabling Z protein to exhibit different conformational states. The C-terminal part harbours the late domains. **(B)** Comparison of OW and NW arenavirus Z proteins. The N-terminal myristoylation site and the YxxL-late domain motif are conserved within both OW and NW arenaviruses. C-terminally located late domains differ between OW and NW virus species both in their number as well as their relative position. Gene bank accession numbers for viral sequences: Lassa virus (LASV, NP_694871.1), Lymphocytic choriomeningitis virus (LCMV, P18541), Lujo virus (LUJV, YP_002929492), Mopeia virus (MOPV, AEO89357), Mobala virus (MOBV, YP_516228), Ippy virus (IPPV, YP_516232), Whitewater Arroyo virus (WWAV, YP_001911119), Pichinde virus (PICV, AAL16100), Pirital virus (PIRV, YP_025092), Junin virus (JUNV, AFA53095), Machupo virus (MACV, AAT45079), Chapare virus (CHAV, YP_001816784),Tacaribe virus (TCRV, NP_694847), Guanarito virus (GTOV, NP_899220), Sabia virus (SABV, YP_089659), Latino virus (LATV, YP_001936025), Oliveros virus (OLVV, YP_001649215).

### 3.1 Structural aspects of the distinct functional properties of Z proteins

While the Z proteins of different arenaviruses vary in their amino acid sequence and length and, in part, their biological functions, they all share distinct structural features and motifs that play important roles in mediating their multifunctional activities. Common characteristics conserved among OW and NW arenavirus Z proteins include an N-terminal myristoylation signal, a central RING domain and small tetra-peptide motifs that function as viral late domains located in the C-terminal arm (Figure 4A).

N-terminal myristoylation is an irreversible, co-translational protein modification during which myristate (a 14-carbon fatty acid) is covalently attached to an N-terminal glycine, catalyzed by the host cell enzyme N-myristoyl transferase using myristoyl-CoA as a substrate upon removal of the initiator methionine by a methionine aminopeptidase [69]. Generally, known functions of the myristyl group include membrane targeting and binding of proteins, enabling protein-protein interaction and providing both the structural basis and conformational stability for the assembly of protein complexes on membranes. N-terminal myristoylation is also a conserved feature of all arenavirus Z proteins that is required for their biological functions [70,71]. The myristyl moiety facilitates Z membrane anchoring and intracellular targeting, Z self-assembly, and interaction of Z with other arenavirus proteins [71,72,73]. Consequently, treatment with myristic acid analogs leads to dose-dependent inhibition of Z-mediated release of VLPs and significantly reduces infectious virus particle production of several OW and NW arenaviruses [70,71,74]. Although the importance of N-myristoylation in Z protein function is well documented, the molecular mechanism(s) by which the myristate acts within the various Z functions is not yet fully understood. Notably, in addition to the N-terminal myristoylation signal, several arenavirus Z proteins contain clusters of basic residues near their N-termini (Figure 4B). As shown for the transforming protein pp60v-src of Rous sarcoma virus, such basic amino acids that are located close to the myristoylation site are required for efficient membrane binding [75]. However, the question of whether N-terminally located basic residues of Z contribute to its membrane association remains to be investigated. Interestingly, structural studies of LASV Z revealed that both the myristoylation signal and the adjacent basic residues are located within a structurally disordered region of Z termed the N-terminal arm (residues 1-29) [76]. This N-terminal arm is highly flexible, thus potentially enabling the basic residues to associate with acidic phospholipids in cellular membranes, which could result in enhanced membrane association of Z. 

RING domains are distinct structural motifs that can be found in many different cellular and viral proteins. They range in length from 40 to 60 amino acids and are structurally well ordered, typically consisting of a single α-helix and multiple β-sheets. Their most prominent feature is the conserved Cys_3_HisCys_4_ amino acid motif, through which they coordinate two zinc cations. With a length of ~60 amino acids and a central Cys_3_HisCys_4_ motif, the highly conserved RING domain of arenavirus Z protein displays all the characteristic features of RING domains (Figure 4A). Structural studies of LASV Z protein revealed that the zinc coordination sites, referred to as site I and II, are located on opposing sides of the RING domain [76]. This arrangement generates structurally different surfaces through which the RING domain is thought to act as a platform for the interaction of Z with its various viral and cellular binding partners. While the arrangement of amino acids around site I is very similar to the structure of known RING domains, the conformation around site II is a characteristic feature currently exclusive to arenavirus Z proteins [76]. It is currently unknown whether this conformation is the basis for some of Z`s unique features. The RING domain (particularly site I) has also been shown to be highly important for correct Z self-assembly, as exemplified by the ability of an isolated LCMV RING domain to form spherical structures *in vitro* [77,78,79]. Such RING domain-mediated super-molecular assembly enhances the biochemical activities of LCMV Z [78]. Whether similar structures are also formed by Z in infected cells remains elusive. Z induces dot-like structures in the cytoplasm of both infected and transfected cells, which are comparable in their dimensions to the structures formed by recombinantly expressed Z protein isolated from bacterial systems. However, due to the lack of detailed structural information of these intracellular assemblies it remains unknown whether they are identical to the spherical structures formed by Z during recombinant protein expression in bacteria. 

Late domains are small tetrapeptide motifs that have been identified in the matrix proteins of various enveloped RNA viruses and in the Gag proteins of a number of retroviruses. They consist of the amino acid sequences P[T/S]AP, PPxY, or YxxL, where ‘x’ represents any amino acid (reviewed in [80]). Late domains mediate protein-protein interactions between viral proteins and components of the endosomal sorting complexes required for transport (ESCRT), which mainly constitute the vacuolar protein sorting (VPS) pathway [80]. Both OW and NW arenavirus species contain a highly conserved YxxL motif located within the central RING domain. Furthermore, all arenavirus Z proteins carry P[T/S]AP- and PPPY-type late domains in their C-terminal parts. However, these vary greatlybetween OW and NW virus species both in their number as well as their relative position (Figure 4B). The Z protein from OW LCMV harbors a PPPY motif and a P[T/S]AP-like domain STAP, while Z proteins from African arenavirus species carry closely spaced a PPPY and a classical PTAP motif. However, the Z protein of the newly discovered OW LUJV is an exception to this rule, and sequence analysis has revealed an additional YxxL motif in place of the otherwise typical PPPY motif. Most NW arenavirus Z proteins contain a P[T/S]AP motif at their C-terminal end. However, TCRV Z shows an ASAP motif at this position. Interestingly, Z proteins from Pichinde virus (PICV), Pirital virus (PIRV), and WWAV possess overlapping PSAP and APPY (a potential PPPY-like late domain) tetrapeptide motifs that share some similarities to the overlapping late domains described for the Ebola virus (EBOV) matrix protein VP40 (PTAPPEY). Notably, the NMR-structure of LASV Z has shown that the C-terminal arm harboring these late domains is very similar to the N-terminal arm in that it, too, is structurally unordered and highly flexible. This flexibility is thought to enable LASV Z to exhibit varying conformational states, thereby allowing the C-terminal arm of LASV Z to mediate interaction with different cellular binding partners through its late domains [81].

In summary, the arenavirus Z protein contains several distinct domains and conserved motifs which, together with its high conformational flexibility, allow Z to interact with various different cellular and viral binding partners.

### 3.2 Z protein as a regulator of virus replication and transcription

Early studies based on immunodepletion of Z proteins from TCRV-infected cell extracts indicated that Z is required for both mRNA synthesis and genome replication [82]. In contrast to these observations, more recent studies using a TCRV minigenome system suggest that Z protein is non-essential for transcription and replication of the viral genome. In fact, co-expression of Z revealed an inhibitory effect on both transcription and replication of the minigenome [68]. Similar results have been observed using a LCMV reverse genetic system, wherein Z was not required for transcription and replication of the LCMV minigenome, but strongly inhibited LCMV minigenome expression in a dose-dependent manner [83]. This negative regulatory role of Z is further supported by the observation that cells that transiently express Z exhibit a significantly decreased susceptibility to infection with LCMV [83], while cells transduced with a recombinant adenovirus that express Z are resistant to infection with LCMV and LASV [84]. Importantly, Z-mediated resistance to superinfection is not due to blocked virus entry, but rather to the strong inhibitory effect of Z on virus polymerase activity, which leads to decreased production of infectious viral particles.

**Figure 5:**
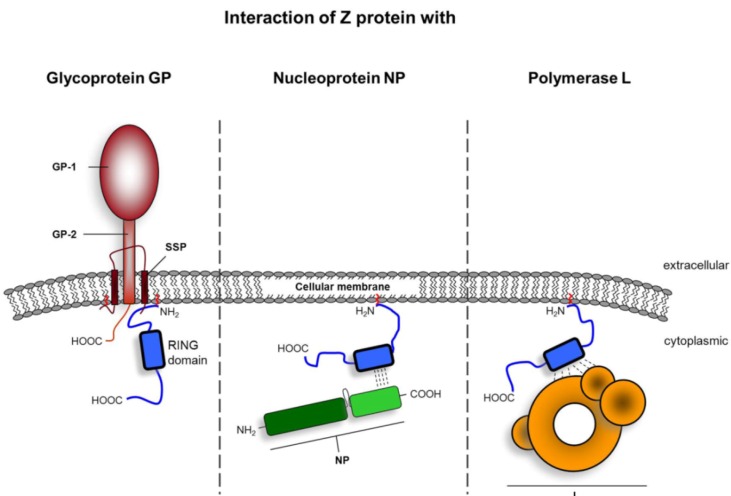
Interaction of Z protein with other arenavirus proteins. The Z protein (blue) coordinates multiple interactions with other viral proteins. Z protein associates with the viral glycoprotein GP (red) through interaction with the stable signal peptide (SSP) of GP. Interaction is dependent on Z myristoylation. Through amino acids located within and adjacent to the RING domain, Z directly interacts with the C-terminal domain of nucleoprotein NP (light green).Interaction between Z and the viral polymerase L (orange) is facilitated by amino acids within the Z RING motif. Z contact sites within L have been mapped to its N-Terminus and the polymerase domain, although the exact locations of the binding sites remain to be determined. The schematic drawing of the polymerase L ultrastructure has been adopted from [86].

Initial clues to the molecular mechanism that Z uses to inhibit the transcription and replication arose from the observation that the TCRV Z protein directly interacts with the L protein (Figure 5) [85], raising the possibility that this interaction might negatively regulate L-driven replication and transcription. L consists of four putative domains [86,87,88], two of which (in the case of TCRV L) have been shown to be binding sites for the Z protein. One binding site was mapped to the N-terminus of L, located between residues 156 and 292. A second binding site was identified within the RNA polymerase domain [87]. Residues within the Z protein that are presumably involved in L binding have been identified by assessing the ability of various Z mutants to inhibit viral RNA synthesis. The inhibitory activity of LCMV and LASV Z requires an intact RING domain, whereas the N- and C-terminal residues are not essential for this function [73,89]. In particular, the Zn^++^-coordinating residues and a highly conserved tryptophan residue within the RING domain are essential for the strong inhibitory activity of Z on RNA synthesis [90]. Furthermore, in the case of TCRV Z, distinct residues located around the Zn^++^-coordinating amino acids have been shown to participate in the direct interaction with the polymerase [73]. Since the L protein still retains its RNA binding properties while associated to Z, the interaction between both proteins is further thought to be important for the process of packaging viral RNA segments into progeny virions during assembly. Additionally, recent studies demonstrated that this direct interaction with L enables Z to inhibit the catalytic activity of the polymerase, thereby rendering it unable to perform viral RNA synthesis [91]. Further to this point, Z may be involved in condensing the nucleocapsid via interaction with NP, thus leading to inhibition of the biosynthetic processes directed by the virus polymerase, as has been shown for matrix proteins of other negative-strand RNA viruses [92,93,94].

### 3.3 The multifunctional activities of the Z protein in virus assembly and budding

#### 3.3.1 The role of the Z protein in glycoprotein GP incorporation

To ensure the recruitment of RNPs to specific areas of the plasma membrane in which GP spikes are concentrated, the production of infectious particles requires a highly organized and well-coordinated process of viral protein-protein interactions during assembly. Since the glycoprotein complex mediates both target cell binding and the subsequent fusion of the viral and host cell membranes during entry, the incorporation of GP into budding particles is essential for facilitating continued infection. The arenavirus glycoprotein is synthesized as an inactive precursor preGP-C that is co-translationally cleaved by signal peptidase into GP-C and the stable signal peptide (SSP) [95,96]. Post-translational maturation cleavage of GP-C by the host cell proprotein convertase S1P (site 1 protease), also known as SKI-1 (subtilisin kexin isozyme-1), then leads to the generation of the distal receptor-binding subunit GP-1 and the transmembrane-spanning fusion competent subunit GP-2 [97,98,99,100]. Since only cleaved subunits are incorporated into budding virions, proteolytic processing of GP-C by S1P is absolutely necessary for the production of infectious particles [98]. Remarkably, the SSP is not only essential for S1P-mediated activation cleavage of GP-C by functioning as a *trans-*acting maturation factor [101], but remains stably associated with GP, primarily through interaction with a zinc-binding domain present in the cytoplasmic tail of GP-2 [102,103,104]. Together with GP-1 and GP-2, SSP forms the tripartite glycoprotein spike complex on the viral surface, making the mature glycoprotein spikes of arenaviruses unique among enveloped single-stranded RNA viruses [50,105].

In spite of the progress made in understanding the structural details of arenavirus glycoproteins, the intracellular trafficking pathway and the maturation of GP, the mechanism by which the tripartite mature GP complex is incorporated into the lipid bilayer of the nascent virions during virus assembly is still only partially understood. The Z protein has been implicated in this process based on observed intracellular interaction between GP and Z (Figure 5) [72,106]. GP and Z are also capable of interacting in VLPs [106], which coincides with their structural organization in arenavirus particles as observed using cryo-electron microscopy [51]. The N-terminal myristoylation of Z is critical for GP-Z interaction, whereas both the RING domain and the C-terminal late domains are non-essential for GP-Z association [72]. Interestingly, Z directly associates with SSP, even in the absence of other subunits of the GP complex [72]. However, the molecular nature of the SSP-Z interaction still remains to be determined. The identification of the protein-protein interaction motifs within SSP and the Z protein will thus provide further insight into the mechanism underlying the incorporation of GP into nascent virions. In particular, defining the precise role of N-myristoylation of Z in this process will be of great interest, as myristoylation of Z has been linked to various biological functions, including intracellular targeting [71]. Suppression of Z myristoylation - either by mutation of the conserved glycine residue at position 2 (Z-G2A), or by treatment of wild-type Z protein expressing cells with myristic acid analogs - adversely affected the subcellular localization of Z, altering it from a characteristic punctuate cytoplasmic distribution pattern to a more diffuse and predominant perinuclear accumulation [71]. In addition, N-myristoylation has been reported to have a strong impact on protein structure [107]. Further studies are therefore required to elucidate whether the limited association observed between GP and Z-G2A is due to altered intracellular localization or to structural changes of mutant Z that perturb efficient GP- Z interaction. 

Association of Z with GP determines directed LASV release from polarized epithelial cells. In dissecting the viral determinants responsible for polarized virus budding, individually expressed Z protein was shown to induce VLP budding from both apical and basolateral surfaces, whereas the addition of GP shifted VLP release to the apical plasma membrane [106]. Accordingly, infectious LASV buds from the apical membranes of polarized kidney cells [106]. 

It is well established that arenavirus particles assemble at the plasma membrane, therefore virion morphogenesis requires GP-Z co-localization at the site of virus budding. However, neither confocal microscopy [106] nor dual-label immuno-gold staining and electron microscopy [108] produced observable GP-Z association at the plasma membrane. Instead, co-localization between these two proteins was observed in vesicle-like structures in the cytoplasm near the nucleus [106], suggesting the potential existence of an intracellular co-trafficking mechanism to virus assembly sites. Although specific viral assembly pathways remain elusive for arenaviruses, data from our laboratory indicate involvement of late endosomal compartments as a trafficking intermediate for LASV Z protein (S.K. Fehling and T. Strecker, unpublished data). Interestingly, for Marburg virus (MARV), a member of the filovirus family, it has been previously reported that the matrix protein VP40 actively recruits the envelope glycoprotein GP to VP40-enriched late endosomal compartments. Accumulation of MARV GP and VP40 in this compartment is assumed to play an important role in the formation of the filoviral envelope and subsequent release of infectious MARV at the plasma membrane [109,110]. It will be interesting to assess whether distantly related arena- and filoviruses share similarities with respect to their intracellular assembly pathways. 

In summary, the collaboration between GP and Z is critical for the production of infectious particles, although the signals and underlying mechanisms for incorporation of glycoprotein spikes into Z-driven virions during assembly remain to be identified.

#### 3.3.2 The role of Z protein in RNP packaging

**Figure 6:**
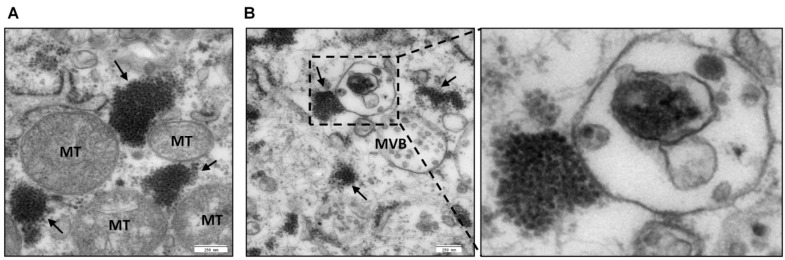
Viral inclusions in LASV-infected cells. Ultrathin sections of LASV-infected Vero cells. Electron micrographs show viral inclusions (arrow) **(A)** near mitochondria (MT) or **(B)** near multivesicular bodies (MVB). Bars, 250nm.

Due to the bi-segmented nature of the arenavirus genome, a highly coordinated and selective packaging mechanism has to ensure that infectious progeny virions contain at least one of each genome segment during assembly. Arenavirus replication takes place in the cytoplasm of infected cells. Cell cultures infected with LASV and other arenaviruses typically reveal distinctive intracytoplasmic electron-dense viral inclusions that are suggested to be important sites for RNA replication (Figure 6) [111,112]. Thus, the RNP complex not only has to be recruited from these cytoplasmic locations to virus budding sites at the plasma membrane, but also requires tethering to budding membranes in order to be incorporated into nascent virions. Early studies using chemical crosslinking have shown that LCMV Z is associated with the viral nucleocapsid in purified particles [53], suggesting that Z is also involved in these processes. Since the RNP complex is composed of NP, L, and genomic RNA, each of these three components could, in principle, harbor the interaction sites that are important for Z-driven RNP packaging. For many other negative-stranded RNA viruses, such as EBOV, interaction between the matrix protein and the nucleoprotein is crucial for nucleocapsid incorporation into nascent virions [113]. Indeed, initial evidence for direct Z-NP interaction in the absence of other viral proteins has already been demonstrated for LASV (Figure 5) [54]. Follow-up studies showed that the intracellular collaboration of NP and Z leads to the incorporation of NP into LASV Z-induced VLPs [106]. Similar findings have been described for NW JUNV and TCRV, as well as for OW LCMV and MOPV [114,115,116,117,118], suggesting that NP-Z association is conserved within the *Arenaviridae* family. Collectively, these data indicate that interaction of NP with Z is important for RNP incorporation during virus assembly. Although recent studies have greatly improved our knowledge of the molecular basis for NP-Z interaction, precise details still remain to be determined. Structural studies of LASV NP and functional analysis of NPs from LASV, LCMV, and TCRV have shown that NP consists of two domains [115,116,119,120]. The N-terminal domain is involved in RNA binding and oligomerization [116,121,122], while the C-terminal domain of NP facilitates type I IFN antagonism [115,119,120,123], oligomerization [121], and association with Z [114,115,116]. Interaction between NP and Z requires an intact RING domain of Z, as it has been shown that mutations of the RING structure drastically affected Z-NP interactions and the ability of JUNV Z to incorporate TCRV NP into budding VLPs [118]. However, future studies are necessary to elucidate whether the functional or structural integrity of the RING domain is of critical importance for the reported observations regarding the role of the RING domain in Z-mediated incorporation of NP into VLPs. Also, mutation of residue L79 to alanine within JUNV Z protein severely impaired intracellular Z-NP interactions and the incorporation of NP into VLPs, resulting in decreased VLP infectivity [118]. This observation has been extended to LASV, since mutation of the corresponding amino acid L71 within LASV Z protein similarly affected the infectivity of recombinant VLPs, supporting the role for Z in the packaging of RNPs into mature infectious virions [89]. Interestingly, for MOPV it has been implied that the host cell protein Alix/AIP1 (ALG-2-interacting protein 1) directly participates in Z-NP interaction, presumably by bridging both proteins [124].

Although the interaction between NP and Z plays a central role in genome packaging, additional mechanism(s) for RNP incorporation may exist. Recent studies have demonstrated that the Z protein also binds to the polymerase L, leading to the formation of Z-L heterodimers [85,91]. While the association between Z and L has been shown to play an important role in regulating viral genome replication and transcription, a recent study by Kranzusch and colleagues suggests that Z-L interaction plays a direct role in ensuring the packaging of the viral polymerase as part of the functional RNP complex [91]. This is particularly important for arenaviruses, since the viral genome per se is not infectious, making polymerase packaging crucial to the production of infectious particles. Interaction with Z renders the polymerase catalytically inactive. Importantly, interaction with Z does not prevent L from binding to viral RNA, as demonstrated by the ability of the Z-L complex to bind to viral promotor sequences [91]. Instead, interaction with Z prevents the early steps of RNA synthesis initiation, therefore keeping the polymerase locked and bound to viral promotor sequences, and hence associated with the viral RNPs. In addition, *in vitro* studies have demonstrated that Z is able to inhibit on-going RNA synthesis, indicating that Z can also contact L during active replication and/or transcription of the viral genome [91]. Whether Z ensures polymerase packaging by interaction of L in the context of a functionally active RNP or whether Z forms a heterodimeric complex with L, which subsequently associates with RNPs through interaction with the viral RNA, remains to be determined.

The matrix proteins of several different RNA virus families, such as bornaviruses, orthomyxoviruses, rhabdoviruses and retroviruses, possess RNA binding capacities, which may have implications for the incorporation mechanism of RNPs into the membrane envelope during budding [92,125,126,127]. The arenavirus Z protein largely binds to nucleocaspids through its interaction with NP, and since the arenavirus NP and L protein are larger and more positively charged than the NP and L of other negative-strand viruses, it is likely that they function as the major RNA-binding proteins. The Z protein central RING domain is a globular type of zinc-binding domains that more frequently binds other proteins, in contrast to zinc-finger domains which are known for intercalating into nucleic acids [128]. Though, the potential RNA binding activities of Z remain to be established. 

Similarly, knowledge of the mechanism underlying intracellular RNP delivery to viral budding zones remains elusive. The functional relationships between Z and NP or Z and L suggest a potential co-trafficking process of the RNP complex via Z trafficking pathways. Consistent with this assumption, the co-expression of Z leads to a redistribution of NP to Z-positive dot-like structures, while solitary expressed NP is evenly distributed in the cytoplasm. Z-NP co-localization was observed either at the plasma membrane, as shown for TCRV [116,118], or within cytoplasmic patches, as documented for LASV, LCMV and MOPV [54,106,114,115]. In contrast, GP seems not to be involved in RNP trafficking since neither a direct interaction nor intracellular co-localization between GP and NP has been detected, which is consistent with the observation that NP is not incorporated into VLPs generated by solitary expression of GP [106]. Co-localization of both proteins in distinct perinuclear clusters was only observed in cells additionally expressing Z protein [106]. Taken together, these observations led to the idea that Z serves as a ‘bridge’ between GP and NP, and therefore links the viral envelope and viral RNP complexes, making Z a key mediator in virus assembly processes. Cryo-electron microscopy of viral particles confirmed that Z is tightly associated with GP and NP, forming a dense layer between both proteins [51]. In a heterologous VLP system using chimeric JUNV/TCRV, the co-expression of TCRV NP greatly enhanced the incorporation of JUNV GP into JUNV Z-induced VLPs, indicating that Z regulates incorporation of GP into nascent virions through interaction with NP [118]. However, when viral protein recruitment was examined in the homologous context, a significant increase in GP incorporation into Z VLPs in the presence of NP was observed neither for NW JUNV and TCRV nor OW LASV [106,117]. 

Altogether, Z protein is a key regulator of virus assembly. Through its interaction with GP, NP and L, the Z protein ensures the incorporation of viral RNP complexes into budding particles that contain glycoprotein spikes, leading to the release of infectious virions. 

#### 3.3.3 Z-mediated recruitment of host cell proteins essential for virus budding

**Figure 7:**
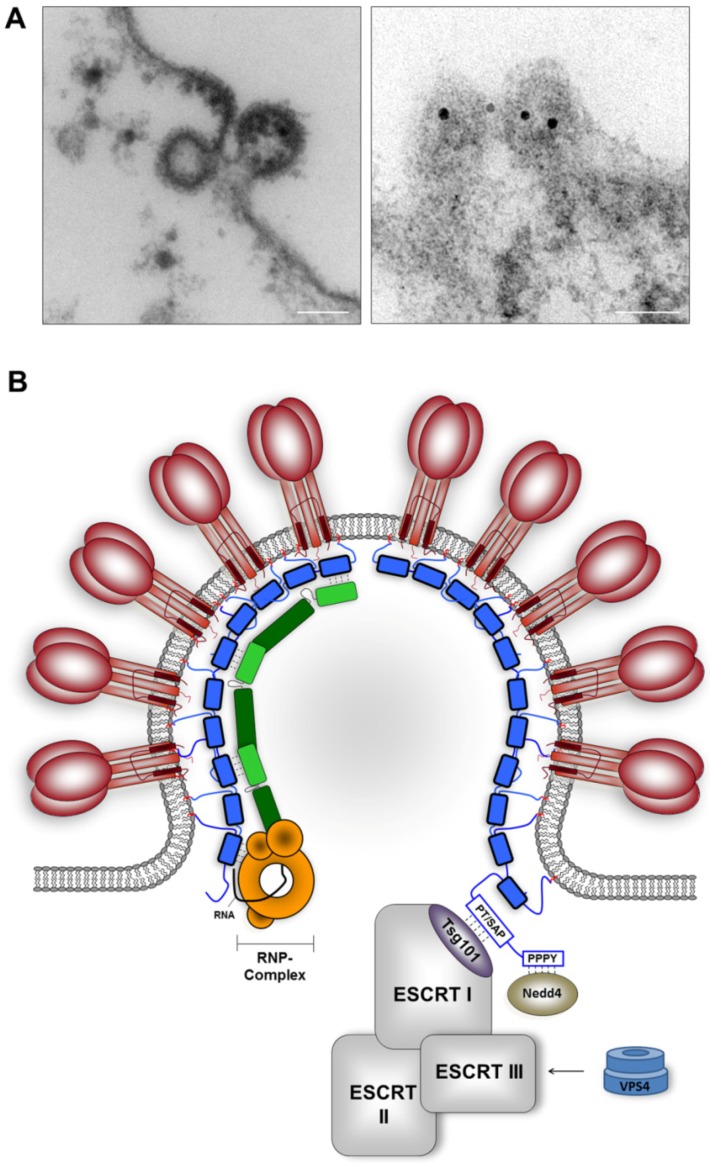
Assembly and budding of arenaviruses. (**A)** Ultrathin sections of LASV-infected Vero cells. The left image shows an electron microscopic picture of LASV budding from the surface of infected cells. Discrete area of membrane thickening and membrane curvature at the left indicates an early stage of virus budding, while the particle shown at the right almost fully assembled. Bar, 100nm. The electron microscopic image shown on the right demonstrates Z protein-specific immuno-gold labeling (10nm gold particles) at LASV budding sites at the plasma membrane. Bar, 50nm. **(B)** Model for arenavirus assembly and budding. Z protein (blue) is the key regulator of virus assembly. Through interaction with GP (red), NP (light and dark green) and L (orange), the Z protein mediates the incorporation of viral RNP complexes into GP-spike containing particles, leading the release of infectious virions from the plasma membrane of infected cells. Viral late domains encoded by Z facilitate interaction with Tsg101 and Nedd4, thereby presumably recruiting other components of the cellular ESCRT machinery. The AAA-ATPase Vps4 is required for efficient budding of arenavirus virions.

Arenaviruses exit their host cells by budding from the plasma membrane (Figure 7A). The final steps prior to virus release involve the envelopment of the viral nucleocapsid by the host cell-derived lipid membrane that harbours mature GP spikes, followed by a subsequent membrane fission event during which the nascent virion is separated from the host cell membrane. Z protein is the key player in these processes. On the one hand, the Z protein mediates all of the essential virion assembly events that ensure the packaging of all viral components required for infectivity, while on the other hand, Z protein alone facilitates virus-host protein-protein interactions that are necessary but also sufficient to create particles (Figure 7B). This assumption is based on the observation that solitary expression of Z protein leads to the formation and release of lipid-enveloped, morphologically regular VLPs at the plasma membrane [52,54,129]. This self-budding activity has been linked to the late domains (Figure 4B) [52,130]. The functional integrity of the late domains is of critical importance for the LASV and LCMV Z-driven release of enveloped particles, since disruption of these motifs abrogated efficient VLP production [52,130]. 

Viral late domains are highly conserved motifs and have been found within viral matrix proteins of numerous enveloped RNA viruses, including rhabdoviruses, filoviruses, and paramyxoviruses as well as within the Gag proteins of various retroviruses (reviewed in [80,131]). Through interaction with individual proteins of the cellular ESCRT complexes, they facilitate the recruitment of the VPS machinery to virus budding sites. The VPS pathway normally functions in the downregulation of cell surface receptors and their sequestering into MVBs, which subsequently deliver their intraluminal cargo for lysosomal degradation. Notably, the sorting of protein cargo into the lumen of the MVB occurs by a vesicular budding event that is catalyzed by the coordinated action of the ESCRT complexes [132,133]. The ESCRT machinery comprises the four multiprotein complexes, ESCRT-0, ESCRT-I, ESCRT-II, ESCRT-III, as well as several ESCRT-associated factors that include Alix/AIP1 and the AAA-ATPase Vps4. ESCRT-0 plays a role in recruitment of ubiquitinated cargos and their concentration at the endosomal membrane. ESCRT-I and II cooperate to induce membrane bud formation and confine cargo, while the ESCRT-III protein complex causes membrane scission that results in the release of vesicles into MVBs [134]. Finally, Vps4 plays an essential role in the completion of vesicle formation by disassembling ESCRT-III components from the target membrane. Because both processes are directed away from the cytosol, ESCRT-driven budding of vesicles into the lumen of the MVB topologically resembles the budding of enveloped particles from the plasma membrane (reviewed in [135,136,137]). It is therefore not surprising that viruses recruit the cellular ESCRT machinery to organize budding of viral progeny, mediated through their late domains. Viral proteins that encode P[T/S]AP late domains gain access to the ESCRT pathway through interaction with the ESCRT-I component Tsg101 (tumor susceptibility gene 101) [138,139,140], while the YxxL motif mediates binding to Alix/AIP1, which is an ESCRT-I and III binding partner [141,142,143]. The PPxY late domain motif has been linked to interactions with various members of the family of mammalian Nedd4 (Neuronal precursor cell-expressed developmentally downregulated 4) E3 ubiquitin ligases (hereafter called Nedd4-like proteins) [144,145,146,147,148,149,150]. 

**Figure 8:**
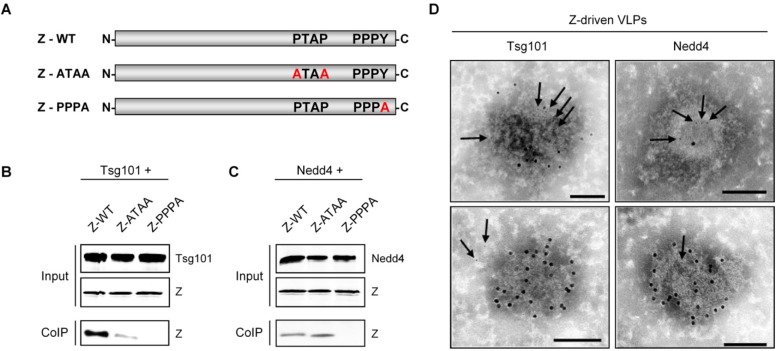
Interaction of LASV Z protein with Tsg101 and Nedd4. **(A)** Wild-type Z protein and substitution mutants used in this study are shown schematically. Co-immunoprecipitation analysis of LASV Z protein and Tsg101 **(B)** or Nedd4 **(C)**. 293T cells were co-transfected with plasmids encoding the indicated Z constructs and Tsg101 or Nedd4. For studies with Nedd4, we used the WW modules (protein-protein interaction domains containing two conserved tryptophan residues) 2-4 of Nedd4 that have been shown to be sufficient for interaction with the EBOV VP40 [194]. At 48h post-transfection, cells were lysed and Tsg101 or Nedd4 were precipitated using protein-specific antibodies. Co-precipitated Z protein was detected by SDS-PAGE with subsequent immunoblotting using a Z-specific antibody. **(D)** Tsg101 and Nedd4 are recruited into Z-induced VLPs. Electron micrographs show Z-driven VLPs that were immunostained for incorporated Tsg101 (left panel, 5nm gold particle, arrows) and Nedd4 (right panel, 5nm gold particle, arrows). Z protein was specifically stained using antibodies coupled to 10nm gold particles. Bars, 100nm.

Tsg101 has been reported to be involved in LCMV and LASV budding. Perturbing Tsg101 function by RNA interference resulted in decreased levels of both LASV and LCMV Z-mediated budding [129,130]. However, the mechanism by which Tsg101 is recruited to virus budding sites is not fully understood. Particularly in the case of LCMV it will be of interest to elucidate the molecular relationship between Z protein and Tsg101, since Z lacks the classical Tsg101-binding motif PTAP. Direct interaction between Tsg101 and Z protein has not yet been established and it remains to be determined whether the late domains within Z play a role in this process. To investigate Z-Tsg101 interaction, wild-type LASV Z protein or Z mutants with individually disrupted PTAP and PPPY late domains were tested for binding to Tsg101 in a co-immunoprecipitation assay. Wild-type Z protein is efficiently co-precipitated with Tsg101 following recombinant expression in human embryonic kidney 293T cells (Figure 8A and B). Mutation of the PTAP motif (ATAA) resulted in a decreased ability of Z to interact with Tsg101, demonstrating that - in the case of LASV - the PTAP motif contributes to, but is not the sole determinant of Z-Tsg101 binding. Remarkably, the PPPY motif appears to be very important for interaction with Tsg101. Mutation of PPPY to PPPA (which has been shown to be sufficient to block VLP release just as effectively as a PPPY deletion mutant [52]) completely eliminated the association between LASV Z and Tsg101 (Figure 8B). Consistent with these observations, the PPPY motif plays a dominant role in Z-mediated budding, whereas the effects of mutating the PTAP late domain were much less severe [52]. A very similar observation has been made for the interaction of Tsg101 with the MARV matrix protein VP40, which was reported to be dependent on the presence of a functional PPPY motif [151]. In contrast, mutations of the PPPY motif within the Gag protein of human T-cell leukemia virus type 1 (HTLV-1) had no effect on its ability to bind Tsg101 [152], suggesting that various viruses have evolved different mechanisms for recruiting Tsg101. 

Because Nedd4 and Nedd4-like proteins have been implicated as critical host factors for PPxY motif-dependent budding, the requirement of a functional PPPY motif indicates that upstream ubiquitin ligase activity may be necessary before Z protein recruits Tsg101. Nedd4 and Nedd4-like ubiquitin ligases are part of an ubiquitination enzyme cascade that involves an ubiquitin-activating enzyme E1 and an ubiquitin-conjugating enzyme E2. The final step mediated by E3 ubiquitin ligases results in the covalent attachment of ubiquitin to target proteins. Ubiquitination often plays a central role in recruitment of proteins by the ESCRT machinery, which may explain also the link between ubiquitin ligases and virus budding [153,154]. The potential involvement of Nedd4 in Z-mediated budding is demonstrated by the observation that LASV Z protein efficiently binds to this host cell protein (Figure 8C). Co-immunopreciptation studies also show that this interaction requires an intact PPPY motif, since the PPPA mutant failed in its ability to bind to Nedd4, while mutations of the PTAP motif have no effect of Z-Nedd4 interaction (Figure 8C). In addition, several Nedd4-like proteins, including WWP1, WWP2, Itch, Smurf1, and Smurf2, are capable of interacting with Z protein (T. Strecker, unpublished data). These observations indicate that Nedd4 or Nedd4-like proteins may link the Z protein to the VPS pathway, although the exact contribution of the individual ubiquitin ligases in Z-mediated budding remains to be determined. However, previous work has shown that overexpression of a dominant-negative mutant of Nedd4 and depletion of Nedd4 by siRNA does not reduce LASV Z-driven VLP production [129]. Notably, human Nedd4 proteins exist in at least two principal isoforms designated Nedd4-1 and Nedd4-2, which are encoded by different genes [155]. Nedd4-1 (but not Nedd4-2) is recruited by HTLV-1 Gag and subsequently facilitates its ubiquitination [149]. More work will be necessary to elucidate the details of the specific role of individual Nedd4 proteins in arenavirus budding. The observation that LASV Z is physically able to interact with Nedd4 and Nedd4-like proteins raises the question of a potential role of ubiquitination of Z and its functional relevance in arenavirus budding. The Z protein of LASV exhibits multiple ubiquitinated forms in cells that also overexpress ubiquitin, indicating that Z protein is a target for ubiquitination. Interestingly, a similar protein pattern of Z was observed in cells that coexpress various Nedd4-like proteins (T. Strecker, unpublished data). Future studies will be aimed at understanding the precise function of ubiquitination in arenavirus budding. 

The molecular details of how the Z-Tsg101 and Z-Nedd4 interactions promote virus budding from the plasma membrane are currently being elucidated in our laboratory. For LASV we propose a model in which Z interacts successively with Nedd4 and Tsg101. Both factors were efficiently incorporated into Z-specific VLPs, supporting the concept of their intracellular interaction (Figure 8D). Interaction with Nedd4 is assumed to lead to ubiquitination of Z and subsequent Tsg101-mediated recruitment of the ESCRT machinery, which then facilitates the budding of virus particles. Tsg101 not only interacts with PTAP late domain motifs, but also recognizes ubiquitin that is attached to proteins [156,157], which could explain the mechanism underlying the participation of Tsg101 in PPxY motif-dependent LCMV Z protein budding. TCRV Z protein buds from cells despite lacking the classical late domain motifs P[T/S]AP and PPxY and does not require Tsg101 activity [158]. Additionally, the potential late domain motifs ASAP and YLCL found in TCRV Z do not play a role in Z self-budding activity [117,158], indicating that TCRV Z employs a budding mechanism distinct from that found in other arenaviruses. The differences in type and number of late domains within various Z proteins are still not fully understood and it is not currently known whether the P[T/S]AP and PPxY motifs act in a redundant or synergistic way in arenaviruses in which both motifs are present. Similarly, further studies are required to address whether the individual late domains act in a cell type-dependent manner. For example, the PSAP motif within the matrix protein of vesicular stomatitis virus was recently reported to contribute to cell type-dependent virion egress [159]. The identification of cell type or host cell-species specific factors will be important to better understand the mechanisms that determine efficient arenavirus budding, and thus viral tropism. In spite of the mechanistic differences between the ways various arenaviruses utilize the cellular VPS pathway for virus budding by recruiting different components of the ESCRT machinery, they all share the requirement of Vps4 for the final release of virus particles from the host cell membrane [129,158]. Although many questions still remain unanswered, current results highlight the important role of Z protein in the final stage of arenavirus budding. In order to better understand the sequence of events that finally lead to the release of infectious arenavirus particles, one of the major future tasks is to identify the individual ESCRT components that are necessary and also sufficient to mediate the budding process. 

### 3.4 Z-mediated regulation of host cell functions

During the viral recplication cycle, the Z proteins of several arenaviruses regulate key host cell functions and favor viral replication, predominantly through direct interaction with different cellular factors. Currently, known cellular factors include the promyelocytic leukemia protein (PML), the nuclear fraction of the ribosomal protein P0, the eukaryotic translation initiation factor 4E (eIF4E) and the proline-rich homeodomain protein (PRH).

PML is an important regulator of mammalian cell growth that forms nuclear bodies which are altered by some disease conditions including acute promyelocytic leukemia and viral infection [160]. In cells infected with LCMV, PML is relocalized from the nucleus to the cytoplasm where it forms large bodies with Z. Furthermore, LCMV and LASV Z proteins alone are sufficient to redistribute PML to the cytoplasm and form specific complexes *in vitro*, implying a direct interaction between these two proteins [161]. Potential functions of the association between Z and PML include evasion of host cell apoptosis (given that PML promotes apoptosis [162,163]), and evasion of the host cell immune response, since PML is assumed to play a role in antiviral defense [164,165].

Ribosomal P proteins (P0, P1 and P2) are essential parts of the large ribosomal subunit and are involved in many of the ribosomal functions, such as binding to 28S RNA and association with eukaryotic elongation factors and aminoacyl tRNA. Studies of LCMV infected cells have shown that a distinct nuclear portion of Z colocalizes with ribosomal P proteins (P0, P1, and P2), which are an essential part of the large ribosomal subunit [166]. However, in contrast to PML, the P proteins are not redistributed upon LCMV infection. Instead, the expression level of P1 and P2 are substantially downregulated. Since P1 and P2 are part of the ribosome, this Z-mediated down-regulation might perturb ribosomal function. In contrast, the level of P0 remains unchanged during infection. The nuclear fraction of P0 has been associated with translationally coupled DNA excision repair and with nonspecific endonuclease activity. Thus, P0 may be involved in the nucleic acid processing activities necessary for LCMV replication, which might explain why its expression level is not affected unlike P1 and P2. Interestingly, Z not only colocalizes, with but also binds directly to the nuclear fraction of P0. Consequently, P0 is also present in LCMV virions, which may explain why LCMV and other arenaviruses contain ribosomes within their virions [166]. However, the exact details of this potential Z-mediated incorporation of ribosomes and its possible functions in the viral life cycle remain unclear.

The eukaryotic translation initiation factor eIF4E is a key component of the cellular translation machinery. It facilitates the binding of ribosomes to the 5` cap structures of cellular mRNAs, hence promoting eIF4E-dependent protein translation [167]. LASV and LCMV Z proteins have been shown to interact with eIF4E. Through this interaction, Z selectively represses protein expression in both infected and transfected cells, and this is thought to favor the establishment of chronic infections [168]. Structural and biochemical studies have revealed that Z does not affect eIF4E expression or stability, but binds directly to the cellular factor [79]. Through residues located within the first zinc-binding site of its RING domain, LASV Z contacts the dorsal surface of eIF4E and induces conformational changes in its distal surface, which itself harbors a cap binding site [76]. In turn, this significantly reduces the affinity of eIF4E for its natural substrate (*i.e*., cellular 5' cap structures), resulting in the specific reduction of protein synthesis dependent on eIF4E [168]. Interestingly, eIF4E-dependent proteins include factors involved in host cell innate immunity, such as IRF-7 (interferon regulatory factor 7), which is a key regulator of the type I IFN-dependent immune response. Therefore, the inhibition of eIF4E through Z may represent an additional mechanism evolved by arenaviruses to counteract the host IFN system (as discussed below).

In specific arenavirus-host contexts, the Z protein may also influence virus replication and pathogenesis, through association with the host factor PRH, a cellular transcription factor involved in the early development of the brain, thyroid, and liver [169]. PRH is specifically expressed in liver cells during liver injury and plays a role in subsequent regeneration processes. By infecting human hepatic cells lines or rhesus macaques with the highly virulent strain LCMV-WE or the related avirulent strain LCMV-ARM, it was found that LCMV-WE infection of liver cells leads to drastic reduction of PRH expression, while the level of expression remains unaltered during infection with the avirulent LCMV-ARM [170]. This is particularly interesting, since LCMV-WE but not LCMV-ARM induces hepatocyte proliferation in infected rhesus macaques [171]. High virus titers are recovered from the liver of infected animals only upon challenge with LCMV-WE [171,172]. Down-regulation of PRH is thus thought to eliminate the antiproliferative effects of PRH and to promote liver cell division, thereby favoring virus replication while leading to pathogenesis by blocking the regeneration of liver cells. Biochemical studies revealed that the LCMV Z protein directly interacts with PRH through its RING domain [170,173], which is why Z is thought to play an important role in the down-regulation of PRH. However, whether this effect is mediated by Z alone, or whether additional viral proteins are involved in the process, is currently not known. Notably, the Z-PRH interaction may play a significant role in specific arenavirus-host contexts but it seems unlikely that it is a general mechanism by which arenavirus infection induces disease, since up-regulated liver proliferation is observed only in rhesus macaques infected with LCMV-WE. In contrast, mice infected with low doses of LCMV-WE showed no signs of liver damage [174]. 

In summary, the arenavirus Z protein interacts with a variety of different cellular factors. Although the exact molecular details of some of the associations between Z and these cellular factors are not fully understood, potential effects include evasion of host cell defense and apoptosis, inhibition of ribosomal functions, and selective repression of host cell protein expression. All these interactions may trigger or counteract a plethora of host cell reactions that favor efficient viral replication or the establishment of chronic infections. The involvement of Z protein in these diverse processes underscores the important role of Z in the arenavirus life cycle. 

### 3.5 New World arenavirus Z proteins antagonize the host cell interferon system

Many different viruses encode for antagonists of the type I IFN response in order to evade cellular immunity. Type I IFNs (IFN-α/β) are secreted mediators in vertebrate immune systems that induce an antiviral state in their target cells, thereby limiting virus replication and spread. With the exception of TCRV, all arenaviruses analyzed to date have been shown to inhibit the up-regulation and production of type I IFNs in cell culture to some extent [175,176,177,178]. Early studies on IFN production in LCMV-infected cells showed that this suppression is caused by a block in the activation of the latent transcription factor IFN regulatory factor 3 (IRF‑3) [179]. In this study, NP was identified to be sufficient for inhibiting both nuclear translocation of IRF‑3 and IFN production. Subsequent work has extended this finding to the NPs from the OW LASV arenavirus and the NW JUNV and MACV arenaviruses, while it was also shown that the TCRV NP is unable to inhibit IFN production [175]. Subsequently, it was found that the activity of NP as an IFN antagonist is based on its ability to digest double-stranded RNA [119,120,123], which is a potent inducer of IFN production. Additionally, different arenavirus NPs directly interact with IKKε (IκB-kinase epsilon), which is an essential component of the IRF-3 signaling pathway. NP-mediated blocking of IKKε activity inhibited downstream signaling, preventing induction of IFN production [180]. Taken together, these observations led to the notion that NP is the main arenavirus interferon antagonist. 

Interestingly, in the case of NW arenaviruses, the Z protein also participates in the suppression of type I IFN production. While NP is supposed to interfere at various steps in the IFN induction cascade, the Z proteins of NW arenaviruses specifically interact with RIG-I (retinoic acid-inducible gene I product) [181], which has an essential function in double-stranded RNA-induced innate antiviral response. Upon RNA binding, RIG-I associates with its interaction partner MAVS (Mitochondrial AntiViral Signaling), leading to the activation of a signaling cascade which results in the nuclear translocation of IRF-3 and hence the production of IFN-β. Importantly, RIG-I has been shown to be involved in the up-regulation of type I IFN expression in response to transfection of cells with LCMV genomic RNA [182]. However, the interaction between RIG-I and Z disrupts the necessary complex formation between RIG-I and MAVS, thereby inhibiting downstream signaling and consequent transcriptional induction of the IFN-β response [181]. This mechanism seems to be specific to the NW arenaviruses TCRV, GOTV, JUNV, MACV and SABV, since Z proteins of LCMV and LASV do not directly interact with RIG-I. Therefore, in the case of NW arenaviruses, both NP and Z may contribute to the inhibition of the type I IFN response.

The importance of the suppression of interferon induction for successful viral replication was recently demonstrated by the observation that the IFN-α induced cellular factor tetherin is able to inhibit arenavirus spread. Tetherin is a membran-associated protein and is understood to function as part of IFN-induced innate immunity against enveloped viruses. It has been shown to prevent HIV-1 release by retaining fully formed progeny virions on the surfaces of infected cells [183]. Importantly, tetherin inhibited both the release of LASV Z-induced VLPs [184] and the egress of infectious LASV and MACV [185]. The potential contribution of NW arenavirus Z proteins to the suppression of the host cell interferon response may result in reduced tetherin expression, thus enabling efficient virus spread.

## 4. Arenavirus Z protein as an antiviral target

Currently, there is no effective antiviral therapy available for combating human pathogenic arenaviruses. Particularly in patients suffering from VHF, the difference between severe out­come and recovery appears to be associated with the viral load [186]. Antivirals that reduce the amount of infectious particles, even transiently, may be effective and may thus have an important impact on the progress of disease. Its multifunctional nature and its important role in the viral life cycle make the Z protein an attractive target for antiviral intervention. As a consequence, recent years have seen great efforts being made in the development of novel approaches that target either the production or function of Z protein.

One such approach is the down-regulation of gene expression through RNA interference (RNAi) using small interfering RNAs (siRNAs) that direct a sequence-specific degradation process of mRNA in mammalian cells. Several reports have demonstrated the efficiency and specificity of synthetic or vector-based siRNAs in the inhibition of arenavirus replication [187,188,189]. Using a recombinant adenoviral delivery system, RNAi-mediated targeting of Z mRNA efficiently inhibited LCMV multiplication in acutely infected cells [187]. Interestingly, the same study showed that RNAi treatment was also effective in clearing persistently LCMV-infected cells. Effective inhibition of virus replication by treating cells with Z-specific siRNAs has also been demonstrated for NW arenavirus JUNV [189]. Taken together, these data demonstrate the feasibility of RNAi targeting of Z protein to achieve inhibition of arenavirus replication. RNAi treatment should thus be given serious consideration as a molecular therapeutic strategy in the fight against arenavirus infections in humans. 

The critical importance of the highly conserved Zn^++^-binding RING domain of Z to viral replication makes this motif an interesting antiviral target. A zinc-reactive disulfide compound (NSC20625) significantly reduced the replication of JUNV, TCRV, and PICV [190]. Treatment of LCMV particles with NSC20625 allowed virions to attach and enter cells, but hindered subsequent viral RNA replication. Extended studies with JUNV revealed that NSC20625 causes a block during the uncoating of viral nucleocapsids from endosomes [191]. Further investigation of the mechanism underlying the antiviral properties of NSC20625 showed that this compound induced unfolding and oligomerization of Z to high-molecular-mass aggregates, presumably through irreversible modifications of the intermolecular disulfide bonds of the cysteine residues in the RING finger domain [192]. Notably, perturbation of the RING domain structure by NSC20625 seems to be specific to arenavirus Z proteins, since cellular RING finger proteins such as PML remain unaffected [192]. Interestingly, NSC20625 blocks interaction between Z protein and PML, thereby suppressing Z-mediated redistribution of PML-formed nuclear bodies [193]. Together, these observations indicate that NSC20625 represents a promising lead compound for the development of a new class of anti-arenavirus inhibitors.

In summary, due to its essential roles in the arenavirus replication cycle, the Z protein represents an important antiviral target. This should pave the way towards the exploration of novel therapeutic strategies for counteracting infection by human pathogenic arenaviruses and especially those that cause life threatening VHF.

## 5. Concluding remarks and future outlook

Great progress has been made in defining both the viral and cellular determinants that are important to the arenavirus life cycle and, in particular, the role of the multiple functions of the Z protein (summarized in Table 1). Z is a critical regulator of replication, the main driving force for the release of infectious progeny, a structural component of virus particles and, an IFN antagonist. Additionally, Z has been implicated in a number of virus-host interactions. The diverse functions of Z are facilitated by a combination of biochemical modification and structural properties including myristoylation, a central RING domain, and late domain motifs that are important to the virus release process. Initial achievements in employing Z as an antiviral target further highlight its critical importance to the viral life cycle. However, a number of key aspects concerning replication, assembly, and budding remain unclear. Urgent questions include: (i) How is Z protein transported to particle assembly sites? Intracellular Z trafficking pathways and the mechanism underlying transport of Z to the site of virus budding are not fully understood. Since the Z protein mediates particle assembly and budding, studies on the intracellular localization of Z should also provide insight into the mechanisms of arenavirus assembly. (ii) When and where does Z protein engage glycoprotein GP and RNPs during the virus assembly process? Although genomic RNA, GP, NP, and L are non-essential for the formation of lipid-enveloped particles, infectious particle production requires their incorporation. (iii) What are the host factors that are involved in the late events of virus release? Arenavirus budding occurs at the plasma membrane, but the preceding steps involved in particle assembly and egress from host cells remain unexplained. These are important areas for future research and will provide critical new information on the cell biology and pathogenesis of arenaviruses. Moreover, a full molecular understanding of how arenaviruses employ cellular resources for their successful replication will open new strategies for the development of novel antivirals in the fight against the severe diseases caused by human pathogenic arenaviruses.

**Table 1:** Summary of Z protein-protein interactions

**Inter-arenaviral protein interactions**					
**Interaction partner**	**Function**		**Domain/motif** **of Z protein**	**Shown for**	**Citations**
**GP**	Z-GP interaction through association of Z with SSP of GP, recruitment of GP into Z-driven VLPs		N-terminal myristoylation	LASV LCMV	[72]
					[106]
**L**	Inhibitory effect on transcription and replication by locking a polymerase-template complex		RING domain conserved W36 residue in LCMV Z	LCMV TCRV	[85]
					[90]
					[91]
	Z-L interaction		RING domain	TCRV	[85]
**NP**	Z-NP interaction, recruitment of NP into VLPs		RING domain Residue L79	JUNV	[118]
	Recruitment of NP into VLPs, enhancement of TCRV Z-mediated budding		ASAP motif	TCRV	[117]
	Recruitment of NP into VLPs, enhancement of TCRV Z-mediated budding		YLCL motif	TCRV	[117]
	NP incorporation into Z-induced VLPs		YLCL motif	MOPV	[124]
**Z**	Association with membranes, intracellular targeting		N-terminal myristoylation	LASV LCMV	[71]
					[70]
	Self-association, multimerization		RING domain, N-terminal myristoylation	LCMV JUNV TCRV	[77]
					[73]
**Virus-host cell protein interactions**						
**Interaction partner**	**Function**	**Domain/motif** **of Z protein**		**Shown for**	**Citations**
**Alix/AiP1**	NP-Z interaction	YLCL motif		MOPV	[124]
**eIF-4E**	Repression of eIF-4E dependent translation	RING domain		LCMV	[168]
**Nedd4**	Exploitation of the VPS pathway for virus release	PPPY motif		LASV	Present study
**P0**	unknown	unknown		LCMV	[166]
**PML**	Relocalization of PML from the nucleus to the cytoplasm and therefore evasion of host cell apoptosis	unknown		LCMV	[166]
**PRH**	Inhibition of the antiproliferative effect of PRH, favoring viral replication and leading to pathogenesis	unknown		LCMV	[170]
**RIG-I**	Inhibition of type-I IFN induction	unknown		JUNV GTOV MACV SABV	[181]
**Tsg101**	Exploitation of the VPS pathway for virus release	PTAP and PPPY motifs		LCMV LASV	[130]
					[129]
					Present study

## List of abbreviations

BSL-4: biosafety level 4
eIF4E: eukaryotic translation initiation factor 4E
ESCRT: endosomal sorting complex required for transport
GP: glycoprotein
GP-C: glycoprotein precursor
IFN: interferon
IKKε: IκB kinase epsilon
IRF-3: interferon regulatory factor 3
JUNV: Junin virus
kDA: kilo Dalton
L: L protein, arenavirus polymerase
LASV: Lassa virus
LCMV: lymphocytic choriomeningitis virus
NF-ĸB: nuclear factor kappa light chain enhancer of activated B cells
Nedd4: neural precursor cell expressed developmentally down-regulated protein 4
NP: nucleoprotein
P0: ribosomal protein P0
PML: promyelocytic leukemia protein
PRH: proline-rich homeodomain protein
RIG-I: retinoic acid inducible gene I
RING: really interesting new gene
RNA: ribonucleic acid
RNAi: RNA interference
SSP: stable signal peptide
TCRV: Tacaribe virus
Tsg101: tumor susceptibility gene 101
VLP: virus-like particle
Z protein: zinc-binding matrix protein
